# *JAK2* Exon 14 Deletion in Patients with Chronic Myeloproliferative Neoplasms

**DOI:** 10.1371/journal.pone.0012165

**Published:** 2010-08-13

**Authors:** Wanlong Ma, Hagop Kantarjian, Xi Zhang, Xiuqiang Wang, Zhong Zhang, Chen-Hsiung Yeh, Susan O'Brien, Francis Giles, Jean Marie Bruey, Maher Albitar

**Affiliations:** 1 Department of Hematology/Oncology, Quest Diagnostics Nichols Institute, San Juan Capistrano, California, United States of America; 2 Department of Leukemia, M.D. Anderson Cancer Center, University of Texas, Houston, Texas, United States of America; 3 The Cancer Treatment & Research Center (CTRC) at the University of Texas Health Science Center, San Antonio, Texas, United States of America; University of Barcelona, Spain

## Abstract

**Background:**

The *JAK2* V617F mutation in exon 14 is the most common mutation in chronic myeloproliferative neoplasms (MPNs); deletion of the entire exon 14 is rarely detected. In our previous study of >10,000 samples from patients with suspected MPNs tested for *JAK2* mutations by reverse transcription-PCR (RT-PCR) with direct sequencing, complete deletion of exon 14 (Δexon14) constituted <1% of *JAK2* mutations. This appears to be an alternative splicing mutation, not detectable with DNA-based testing.

**Methodology/Principal Findings:**

We investigated the possibility that MPN patients may express the *JAK2* Δexon14 at low levels (<15% of total transcript) not routinely detectable by RT-PCR with direct sequencing. Using a sensitive RT-PCR–based fluorescent fragment analysis method to quantify *JAK2* Δexon14 mRNA expression relative to wild-type, we tested 61 patients with confirmed MPNs, 183 with suspected MPNs (93 V617F-positive, 90 V617F-negative), and 46 healthy control subjects. The Δexon14 variant was detected in 9 of the 61 (15%) confirmed MPN patients, accounting for 3.96% to 33.85% (mean  = 12.04%) of total *JAK2* transcript. This variant was also detected in 51 of the 183 patients with suspected MPNs (27%), including 20 of the 93 (22%) with V617F (mean [range] expression  = 5.41% [2.13%–26.22%]) and 31 of the 90 (34%) without V617F (mean [range] expression  = 3.88% [2.08%–12.22%]). Immunoprecipitation studies demonstrated that patients expressing Δexon14 mRNA expressed a corresponding truncated JAK2 protein. The Δexon14 variant was not detected in the 46 control subjects.

**Conclusions/Significance:**

These data suggest that expression of the *JAK2* Δexon14 splice variant, leading to a truncated JAK2 protein, is common in patients with MPNs. This alternatively spliced transcript appears to be more frequent in MPN patients without V617F mutation, in whom it might contribute to leukemogenesis. This mutation is missed if DNA rather than RNA is used for testing.

## Introduction

Myeloproliferative neoplasms (MPNs) are multipotent hematopoietic stem cell disorders characterized by uncontrolled proliferation of maturing blood cells. Chronic myeloid leukemia (CML) is the most common MPN, followed by polycythemia vera (PV), essential thrombocythemia (ET), and idiopathic myelofibrosis (IMF) [1]. Whereas CML is characterized by a readily detectable translocation (Philadelphia chromosome), non-CML MPNs lack recurrent chromosomal anomalies. However, a specific molecular abnormality, the *JAK2* V617F mutation, has been reported in about 95% of PV patients, 35% to 70% of ET patients, and 50% of IMF patients [2]–[4]. *JAK2* exon 12 mutations, as well as other mutations in exons 13 and 14, have been reported in rare cases of non-CML MPDs negative for V617F [5]–[7].

Most testing for *JAK2* mutations is performed by analyzing the genomic DNA of the *JAK2* gene. We have adapted the use of mRNA as the basis for testing for *JAK2* mutations and have shown that RNA allows more sensitive detection of mutations than does DNA at early stages of disease [7]–[9]. The use of RNA rather than DNA provides the additional advantage of capturing abnormalities in platelets and detecting alternatively spliced transcripts.

In a previous report of *JAK2* mutation profiles in a series of >10,000 patient samples, we described detection of a novel deletion of *JAK2* exon 14 (Δexon14) along with other *JAK2* mutations in exons 12 through 15, using bi-directional mRNA sequencing technology [7]. The Δexon14 mutation was detected by direct sequencing in less than 1% of patients with various types of *JAK2* mutations. However, direct sequencing is not amenable to detecting deletions of entire exons, owing to difficulty in interpreting results as well as the low sensitivity of this approach. Therefore, in this study we used a sensitive RT-PCR–based assay with fluorescent fragment analysis to explore the possibility that MPN patients may commonly express this *JAK2* mRNA splice variant at levels that cannot be reliably detected by sequence analysis. We further sought to determine whether patients expressing this splice variant also express a corresponding truncated JAK2 protein.

## Methods

### Patients and Samples

We tested peripheral blood samples from three groups of patients in addition to healthy normal control subjects. Group 1 comprised 61 consecutive randomly selected patients with confirmed non-CML MPN on the basis of clinical findings and complete peripheral blood and bone marrow analysis. The diagnosis of these patients was myelofibrosis in 27 (43%), polycythemia vera in 12 (19%), essential thrombocythemia in 6 (10%) and not-otherwise classified in 16 (27%). The other 2 patient groups were constructed from 183 residual de-identified samples from individuals with suspected non-CML MPNs initially submitted to Quest Diagnostics Nichols Institute for testing of *JAK2* V617F as well as mutations in *JAK2* exons 12 and 13: Group 2 comprised 90 samples that were negative for *JAK2* mutations in V617 and exon 12 and 13, and group 3 comprised 93 samples from patients with *JAK2* V617F. In addition, we tested 46 normal healthy control individuals.

Plasma was separated from peripheral blood samples and used for extraction of total RNA. The mRNA was then used for detection of the *JAK2* Δexon14 variant by RT-PCR with bidirectional sequencing. All samples were also screened for the Δexon14 transcript using a sensitive assay based on RT-PCR with fluorescent fragment analysis.

### Ethics statement

All work was performed according to a protocol approved by an Institutional Review Board (IRB) (Independent Review Consulting Inc. San Anselmo, California). Samples collected from group 1 and the normal control were collected with consent form.

### Sequence Analysis

Total nucleic acid was extracted from patient plasma or PB/BM cells using the NucliSens (BioMerieux, Durham, NC) extraction kit. The primer pair was designed to encompass *JAK2* exons 12 through 14 and part of exon 15: 5′-CTAAATGCTGTCCCCCAAAG-3′ (forward); and 5 ′-CCATGCCAACTGTTTAGCAA-3′ (reverse). The RT-PCR was performed using Superscript III one-step RT-PCR systems with Platinum Taq (Invitrogen, Carlsbad, California) under the following thermocycler conditions: initial step of 94°C for 2 minutes, followed by 40 cycles of 94°C for 15 seconds, 60°C for 30 seconds, and 68°C for 1 minute, with a final extension step of 68°C for 7 minutes. The 491-bp PCR product was then purified and sequenced in both forward and reverse directions using an ABI PRISM 3730XL Genetic Analyzer (Applied Biosystems, Foster City, CA). Sequencing data were base-called using sequencing analysis software and assembled and analyzed with SeqScape software (Applied Biosystems) using GenBank accession number NM 004972 as reference.

### Detection of *JAK2* Δexon14 Transcript Using Fragment Length Analysis

Total nucleic acid was extracted as described above from patient plasma or cells (peripheral blood or bone marrow). The primer pair was designed to encompass *JAK2* exon 14, with the forward primer annealed in *JAK2* exon 13 (5′-GAC TAC GGT CAA CTG CAT GAA A-3′) and the reverse primer annealed in exon 16 (5′-CCATGCCAACTG TTTAGCAA-3′). One of the two primers was FAM-labeled. The RT-PCR was performed using same buffer system (Invitrogen) and thermocycler conditions as the sequencing method. The *JAK2* wild-type and Δexon 14 products were verified by determining the size of PCR products using the GeneScan 350ROX size standard (Applied Biosystems) and ABI PRISM 3730XL Genetic Analyzer. The wild-type product displays a 273-bp peak while the Δexon 14 splice variant displays a 185-bp peak (ie, 273–88 bp). The percentage of transcript accounted for by the *JAK2* Δexon 14 splice mutant is calculated using the following formula:



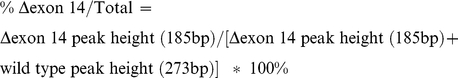



### JAK2 Immunoprecipitation

Cells (5×10^5^–1×10^6^) were lysed in isotonic lysis buffer (150 mM NaCl, 20 mM Tris/HCl [pH 7.4], 0.3% Nonidet P-40, 12.5 mM β-glycerophosphate, 2 mM NaF, 200 µM Na3VO4, and 1 mM phenylmethylsulfonyl fluoride) containing 1× protease inhibitor mix (Roche Applied Science, Indianapolis, IN). Clarified lysates were subjected to immunoprecipitation using TrueBlot™ beads (Ebioscience, San Diego, CA) with an N-terminal anti-JAK2 antibody (JAK2-M126; Santa Cruz Biotechnology, Santa Cruz, CA). After incubation at 4°C for 4 to 12 hours, immune complexes were washed 4 times in lysis buffer, separated by SDS/PAGE, and analyzed by immunoblotting using C-terminal JAK2 antibody (JAK2 [D2E12]; Cell Signaling Technology, Danvers, MA) or N-terminal JAK2 antibody (JAK2 N-17; Santa Cruz Biotechnology). K562 cell line (ATCC CCL-243 Manassas VA) was used as negative control.

### Immunoblot Analysis

Immunoblot analysis was performed as previously described (Bruey et al, Leuk Res. 2009). Briefly, equal amounts of immunoprecipitation products were separated by sodium dodecyl sulfate-polyacrylamide gel electrophoresis (SDS-PAGE), and the gels were electrophoretically transferred to nitrocellulose membranes (0.2-mm pore size; Whatman, Florham Park, NJ). The blots were blocked with 5% bovine serum albumin in Tris-buffered saline with 0.05% Tween-20 (TBS-Tween) for 2 hours. The membrane was incubated with primary antibody for 5 hours at 4°C, washed with TBS-Tween, and incubated with secondary antibody for 30 minutes at room temperature (TrueBlot™, Ebioscience). After additional washing in TBS-Tween, chemiluminescent reagent (ECL; GE Healthcare, Piscataway, NJ) was added and the image was developed on x-ray film.

## Results

### Detection and Prevalence of *JAK2* Δexon 14 Transcript

**Figure 1:**
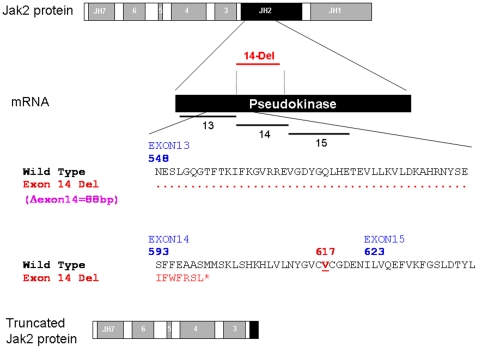
Schematic presentation of *JAK2* Δexon 14. Top, schematic diagram of the JAK2 protein showing JAK homology domains 1 through 7 (JH1-JH7) with the JH2 pseudokinase domain highlighted in black. The corresponding exon regions of the mRNA is shown with the exons 13, 14, and 15. Because exon 14 is consists of 88 bp, its deletion leads to frameshift and early termination of translation after coding for seven new amino acids and elimination of the V617 codon of *JAK2* (lower panel). The resulting truncated JAK2 protein is shown on the bottom.

**Figure 2:**
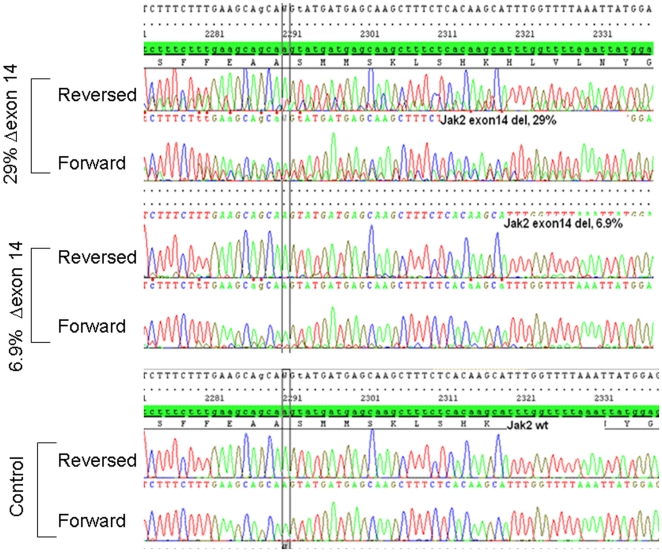
Detection of the *JAK2* Δexon14 transcript with direct bi-directional sequencing. Upper panels: Detection of Δexon14 is relatively easy when the transcript is present at high levels (eg, 29% of total *JAK2* transcript). Detection is more difficult when the Δexon14 transcript makes up a small proportion of total *JAK2* transcript (eg, 6.9%). Normal control is shown on the bottom.

**Figure 3:**
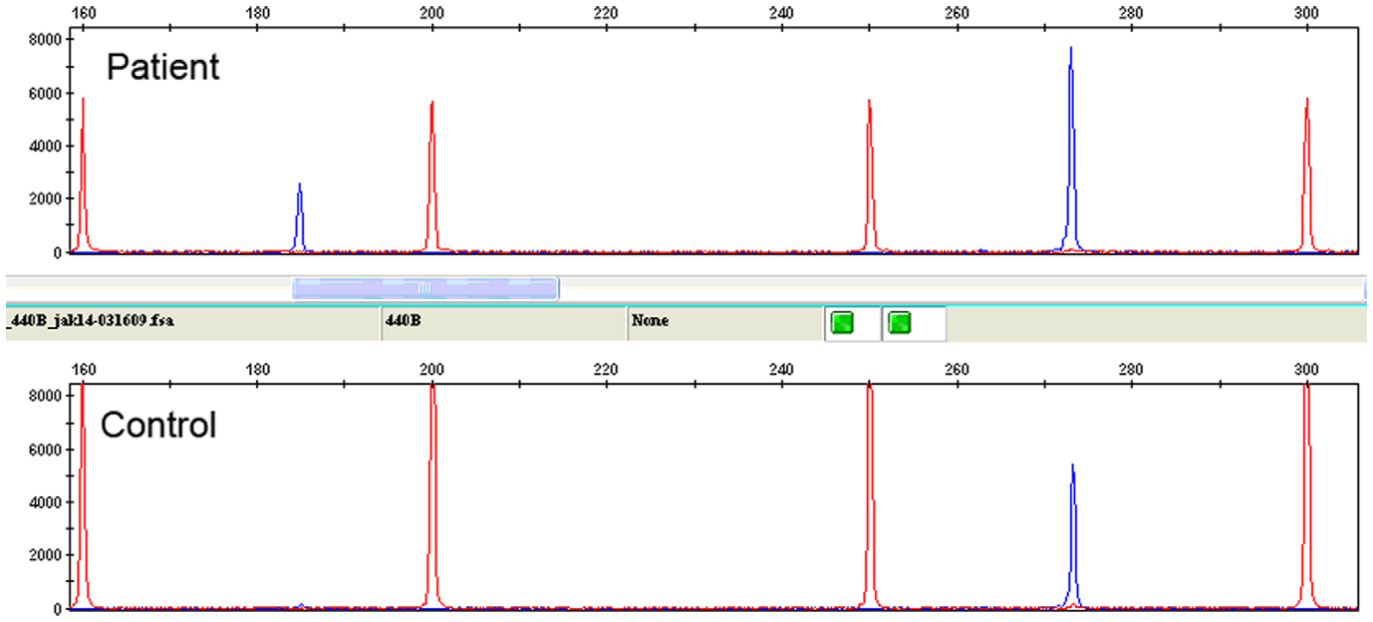
*JAK2* Δexon14 transcript as detected using RT-PCR with fragment length analysis. Lower panel: the expected 273-bp, full-length amplification products; upper panel: the expected 273-bp, full-length wild-type peak in addition to a peak at 185 bp corresponding to the truncated Δexon14 transcript. Size marker is shown as red peaks and amplification products are shown in blue.

**Figure 4:**
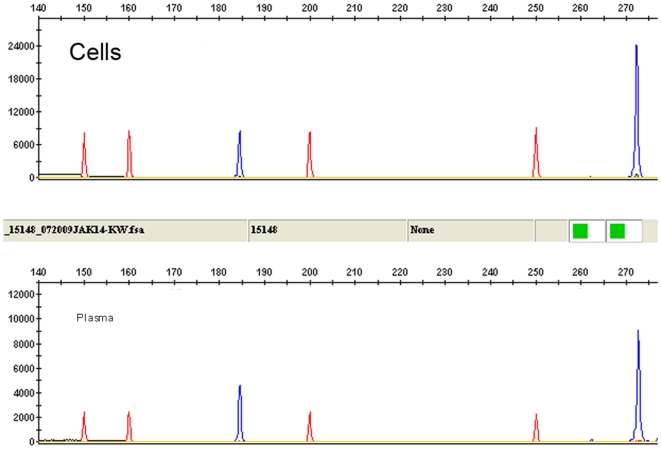
Source of RNA for detection of *JAK2* Δexon14 transcript using RT-PCR with fragment length analysis. Similar results are obtained whether RNA is isolated from plasma or peripheral blood cells. Size marker is shown as red peaks and amplification products are shown in blue.

When *JAK2* RNA (not DNA) is used for direct sequencing, the Δexon 14 transcript is reliably detected if present at levels >15% to 20% of total *JAK2* RNA (Figure 1 and 2). MPN patients rarely have Δexon 14 transcript levels above this threshold, and the results of direct sequence analysis can be difficult to interpret in patients with apparent low-level expression. In these cases, the Δexon14 transcript can easily be interpreted as background or poor sequencing if the background sequence is not read completely and aligned to the *JAK2* sequence (Figure 2). To more accurately detect low levels of Δexon 14 transcript expression, we developed an RT-PCR–based assay with fluorescent fragment length analysis. With this method, the *JAK2* Δexon14 splice variant shows a 185-bp fragment while the wild-type shows a 273-bp fragment (Figure 3). To confirm that the Δexon14 splice variant that is detected in plasma is actually present in cells, we analyzed paired cell and plasma RNA from patients previously confirmed to show expression of the Δexon14 transcript in plasma. Both plasma and cells revealed reliable results for detecting Δexon14 transcripts (Figure 4).

Using the RT-PCR/fragment length analysis assay, we screened all groups of patients. Samples with Δexon 14 mutant to wild-type ratios greater than 15% by fragment analysis were tested and successfully confirmed by careful inspection of direct sequencing results. The Δexon14 was detected in 9 of the 61 confirmed MPN patients (15%) (Table 1), where it accounted for 3.96% to 33.85% (mean  = 12.04%) of *JAK2* transcript. In this group of patients, Δexon14 was detected in 33.3% of V617F-positive patients and in 57.9% of V617F-negative patients. This mutation was also detected in 51 of the 183 patients with suspected MPNs (27%) overall, including 20 of the 93 (22%) V617-positive patients (mean [range] expression  = 5.41% [2.13%–26.22%]) and 31 of the 90 (34%) V617F-negative patients (mean expression  = 3.88% [2.08%–12.22%]. While the difference in prevalence is not statistically significant (*P* = 0.07), there is a tendency of finding Δexon14 in unmutated patients. None of the 46 plasma RNA samples from the normal control group showed any expression of the Δexon14 transcript. Most patients with Δexon14-positive in each group had expression levels below 15%, therefore, it is frequently missed by direct sequencing.

**Table 1:** Prevalence and Relative Level of the ΔExon14 *JAK2* Transcript in Patients with Suspected or Confirmed Myeloproliferative Neoplasms (MPNs)

	Suspected MPN (n = 183)				Confirmed MPN (n = 61)	
	V617F-Negative (n = 90)		V617F-Positive (n = 93)			
	n (%)	Mean (range) percentage of *JAK2* transcript	n (%)	Mean (range) percentage of *JAK2* transcript	n (%)	Mean (range) percentage of *JAK2* transcript
Δexon14	31 (34)	3.88 (2.08–12.22)	20 (22)	5.41 (2.13–26.22)	9 (15)	12.04 (3.96–33.85)
No Δexon14	59 (66)	NA	73 (78)	NA	52 (85)	NA

### Effects of Δexon14 on JAK2 Protein

**Figure 5:**
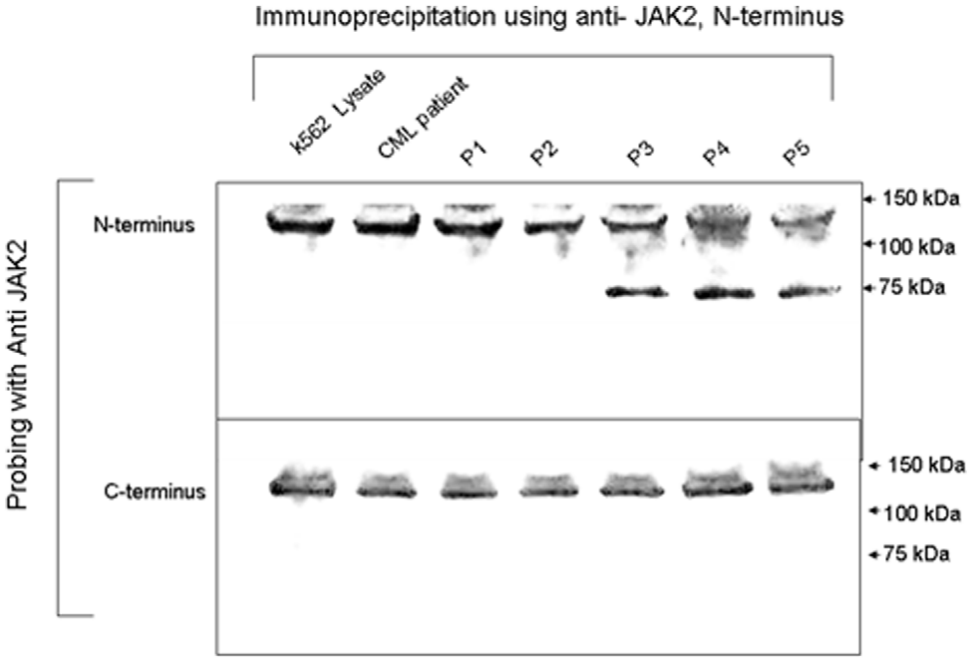
Truncated JAK2 protein resulting from *JAK2* Δexon14 mutation in patients with chronic myeloproliferative neoplasms. Lysates were prepared from the indicated human CML K562 cell line (Lane1), a patient with chronic myelogenous leukemia (lane 2), and 5 patients with chronic MPNs (Lanes 3–7). Patient 1: non-CML CMPD, *JAK2* V617F positive; Patient 2: non-CML CMPD, *JAK2* V617F negative; Patient 3: non-CML CMPD, *JAK2* Δexon14 positive; Patient 4: non-CML CMPD, *JAK2* Δexon14 positive; Patient 5: non-CML CMPD, *JAK2* Δexon14 positive. **Top Panel:** Probing with an anti-JAK2 N-terminal clone yielded a wild-type JAK2 band at 130 kDa in the K562 and other negative control lanes, and an additional band at 75 kDa only in patients with expression of Δexon14 transcript. **Bottom Panel:** An anti-JAK2 clone directed against the carboxyl-terminus of JAK2 yielded only a single band at 130 kDa.

As indicated in Figure 1, deletion of exon 14 leads to a complete deletion of codon V617, a hot spot for mutation in patients with non-CML MPNs [3], [4]. More importantly, since exon 14 is composed of 88 bp, its deletion leads to a frameshift and the coding of new amino acids. However, the frame shift results in the addition of only 7 new amino acids, followed by a termination codon leading to truncation of the JAK2 protein within the pseudokinase domain. Therefore, we used immunoprecipitation and immunoblotting to confirm that the truncated JAK2 protein is expressed in cells from patients with confirmed *JAK2* Δexon14 transcripts (Figure 5). Total JAK2 protein was immunoprecipitated using anti-JAK2 (N-terminal) antibody. For negative controls we used the K562 CML cell line, which does not express JAK2 Δexon14, in addition to cell samples from 1) a patient with CML; and 2) two patients with MPN who had been confirmed by RT/PCR to be negative for Δexon14 transcripts. Cells from three patients with different levels of Δexon14 transcript expression, as confirmed by RT/PCR, were used as positive samples.

Probing with the anti-JAK2 N-terminal clone yielded a wild-type JAK2 band at 130 kDa in the K562 and other negative control lanes, and an additional band at 75 kDa only in patients with confirmed expression of the Δexon14 transcript (Figure 5). This additional immunoprecipitation product represents the truncated JAK2 protein. The use of another anti-JAK2 clone directed against the carboxyl-terminus of JAK2, which is deleted from the truncated JAK2 protein, yielded a single band at 130 kDa, showing specificity of detection of the truncated JAK2 Δexon14 protein (Figure 5). Lysates from the K562 cell line and the CML patient showed only the wild-type band at 130 kDa. This confirms that the Δexon14 transcripts are translated.

## Discussion

Our findings confirm that many individuals with non-CML MPNs, especially those lacking V617F, express low levels of the *JAK2* Δexon14. The paucity of reports of the Δexon14 variant in MPN patients most likely derives from the fact that *JAK2* mutation assays typically rely on DNA rather than RNA. Furthermore, this abnormality cannot be detected with methods that rely on use of specific probes. While bi-directional sequencing of mRNA transcript can detect most mutations, including splice variants, this approach lacks sensitivity (Figure 2). Therefore, a fluorescent fragment analysis method such as the one describe here must be used to detect low levels of this variant. The fact that the *JAK2* Δexon14 transcript is a fraction of the total *JAK2* transcript is, most likely, due to either alternative splicing or a mutation in the DNA in small fraction of the clone. Further studies are needed to fully determine the mechanism of this phenomenon. We did not sequence the DNA in cases with 100% *JAK2* Δexon14 transcript and the possibility of a mutation in the DNA in these patients is likely and should be ruled out.

The fact that the *JAK2* Δexon14 is detected only in patients with MPNs, and more likely in patients negative for V617F (57.9% vs. 33.3%)(*P* = 0.07), suggests that it may play a significant role in the pathophysiology of MPNs. Current knowledge of JAK2 functional domains and known mutations may shed light on the potential biological effects of this unique splice variant. All currently identified *JAK2* mutations, including point mutations and indels in exons 12 through 15, reside in the pseudokinase domain (JH2)-coding region of *JAK2* and do not affect the reading frame [5]–[7]. The JH2 domain is usually in close proximity to the kinase domain (JH1), inhibiting its activation if not bound to an active receptor [10]–[13]. Mutations in the JH2 domain may thus lead to constitutive activation of the JAK2 protein; it is believed that mutations in the JH2 domain cause constitutive activation in the downstream JAK2-STAT signaling pathways as long as the JAK2 is bound to the receptor [14]. Deletion within the JH2 domain and complete deletion of the kinase domain (JH1) is unexpected and raises questions on its mechanism in activating the JAK2-STAT pathway. The Δexon14 mutation preserves the JAK2 FERM domain (JH4-7), which is responsible for association with growth factor (eg, erythropoietin and thrombopoietin) receptors [15]. It is thus possible that the truncated JAK2 dimerizes with wild-type JAK2 to influence its structure, activating its kinase domain and the JAK2-STAT pathway. It is also possible that the Δexon14 mutation causes activation of STAT5 by altering receptor binding of the FERM domain. Clearly, further studies are needed to understand the mechanism by which the truncated JAK2 is involved in any activation of the JAK2-STAT pathway. Ex-vivo or in-vivo experiments showing the effects of expressing *JAK2* Δexon14 transcripts on tumorogenesis should be performed.

In conclusion, the *JAK2* Δexon14 splice variant is a relatively common anomaly in patients with MPNs, possibly more so in those lacking the V617F mutation. Further functional analyses of the Δexon14 JAK2 protein are needed to better understand how this abnormality may contribute to the pathophysiology of disease and its potential role as a marker for diagnosis, prognosis, or prediction of response to therapy.
